# Intradialytic Hemodynamic Instability and Delirium in Critically Ill Patients Receiving Renal Replacement Therapy: A Retrospective Cohort Study

**DOI:** 10.3390/brainsci16101091

**Published:** 2026-10-10

**Authors:** Erdem Baran

**Affiliations:** Department of Nephrology, Yalova Training and Research Hospital, Baglarbasi Mah., Yakup Acar Cad., No:32, Yalova 77200, Turkey; drerdembaran@hotmail.com

**Keywords:** mechanical ventilation, critical care, delirium, renal replacement therapy, intradialytic hypotension

## Abstract

**Highlights:**

**What are the main findings?**

- Patients with CAM-ICU-defined delirium had greater cumulative intradialytic hypotensive burden; however, these differences were no longer statistically significant after normalization to the number of RRT sessions.
- Intradialytic hemodynamic parameters did not show statistically significant associations with delirium after adjustment for age, sex, and admission SOFA score.

**What are the implications of the main findings?**

- Cumulative intradialytic hypotensive burden should be interpreted cautiously, as differences may partly reflect unequal opportunities for exposure accumulation across RRT sessions.
- Prospective studies with standardized hemodynamic monitoring and temporally detailed delirium assessment are needed to clarify the relationship between intradialytic hypotension and delirium.

**Abstract:**

**Background:** Acute kidney injury (AKI) requiring renal replacement therapy (RRT) is common in critically ill patients and is frequently accompanied by delirium. Intradialytic hemodynamic instability, particularly hypotension, may impair cerebral perfusion; however, its quantitative relationship with delirium remains unclear. This study investigated the association between intradialytic hemodynamic instability and Confusion Assessment Method for the Intensive Care Unit (CAM-ICU)-defined delirium in critically ill patients receiving RRT. **Methods:** This single-center retrospective study included 87 adult intensive care unit (ICU) patients undergoing RRT between September 2025 and May 2026. Delirium was assessed using the CAM-ICU. Intradialytic hemodynamic burden was quantified by the cumulative duration of mean arterial pressure (MAP) < 65 mmHg and the number of hypotensive episodes during RRT. Multivariable logistic regression analyses adjusted for age, sex, and admission SOFA score were performed to evaluate the associations between hemodynamic parameters and delirium, while receiver operating characteristic (ROC) analyses were used to assess their discriminative ability. **Results:** Of 87 patients, 46 (52.9%) were classified as CAM-ICU-positive for delirium. Patients with delirium had a longer cumulative duration of MAP < 65 mmHg (median, 36.0 vs. 5.0 min; *p* = 0.048) and more hypotensive episodes (median, 3.0 vs. 1.0; *p* = 0.032). These differences were no longer significant after normalization to the number of RRT sessions (*p* = 0.153 and *p* = 0.249). In addition, neither parameter showed a statistically significant association with delirium after adjustment for age, sex, and admission SOFA score. ROC analysis demonstrated limited discriminative ability (AUC, 0.62 and 0.63, respectively). Mechanical ventilation duration, ICU length of stay or ICU mortality did not show a statistically significant association with CAM-ICU positivity in the adjusted models. Sensitivity analyses using alternative functional forms for cumulative MAP < 65 mmHg duration, including log-transformed, categorical, and natural cubic spline models, did not show statistically significant associations with CAM-ICU positivity. **Conclusions:** Critically ill patients with delirium had greater cumulative intradialytic hypotensive burden; however, these differences were attenuated after accounting for the number of RRT sessions, and the hemodynamic parameters did not show statistically significant associations with delirium in the adjusted regression models. These findings suggest a potential relationship between intradialytic hemodynamic burden and delirium but do not establish an independent or causal association.

## 1. Introduction

Delirium is one of the most common acute neurocognitive disorders encountered in critically ill patients and is particularly relevant among those requiring mechanical ventilation, in whom sedation, systemic inflammation, impaired cerebral perfusion, and multiorgan dysfunction may interact to increase vulnerability to delirium.

Acute kidney injury (AKI) is a major clinical complication, developing within the first 48 h in nearly 50% of critically ill patients admitted to intensive care units [1]. AKI, which can lead to the need for renal replacement therapy (RRT) in some patients, is also associated with a significant increase in hospital mortality [2,3].

Delirium is an acute disturbance of consciousness and cognitive function with a fluctuating clinical course, reported to occur in approximately 40–80% of critically ill patients [4]. Its development has been associated with adverse clinical outcomes, including longer hospital stays, increased mortality, prolonged mechanical ventilation, and long-term neurocognitive impairment [5]. In patients with AKI, several pathophysiological mechanisms may increase vulnerability to delirium, including accumulation of uremic toxins, systemic release of proinflammatory cytokines, alterations in blood–brain barrier permeability, and activation of neuroinflammatory pathways [6,7]. This interaction, in which renal dysfunction triggers central nervous system disturbances, has been described as “kidney–brain crosstalk” and is thought to reflect a broader pattern of multi-organ dysfunction during critical illness [2,8,9,10]. Consistent with this framework, previous studies have demonstrated that AKI is independently associated with delirium in critically ill patients [8,9]. Nevertheless, AKI often occurs alongside other critical conditions—sepsis, shock, mechanical ventilation, sedative exposure, and vasopressor therapy—that may independently contribute to delirium or interact with one another to further increase risk [10]. In critically ill patients, delirium can be systematically assessed using the Confusion Assessment Method for the Intensive Care Unit (CAM-ICU), a validated tool with high diagnostic accuracy in both mechanically ventilated and non-mechanically ventilated patients [11,12].

RRT is a life-saving intervention for severe AKI, particularly when accompanied by significant electrolyte or acid–base abnormalities. However, intradialytic hemodynamic instability, particularly hypotension, is common during RRT and may necessitate adjustments in ultrafiltration rate or initiation or escalation of vasopressor support [13].

Cerebral blood flow depends on systemic arterial pressure and intact cerebral autoregulation. In critically ill patients, cerebral autoregulation may be impaired by several factors, including sepsis, systemic inflammation, cerebrovascular disease, sedative use, and mechanical ventilation. Consequently, reductions in mean arterial pressure (MAP) during RRT may compromise cerebral perfusion and potentially contribute to neurological dysfunction, particularly in patients with impaired autoregulatory capacity [14,15,16]. Importantly, the hemodynamic burden of hypotension may depend not only on its occurrence but also on its severity, frequency, and duration. Recurrent hypotensive episodes and prolonged cumulative exposure to MAP < 65 mmHg may have different physiological consequences from a single transient reduction in blood pressure. Therefore, characterizing hypotension solely as a binary event may not fully capture the hemodynamic burden potentially relevant to cerebral perfusion [17].

Although the association between AKI and delirium has been extensively investigated, the relationship between intradialytic hemodynamic instability and acute delirium in critically ill patients receiving RRT remains insufficiently characterized. In particular, studies examining the association between quantitative measures of hypotensive burden (e.g., the number of hypotensive episodes and cumulative duration of MAP < 65 mmHg) and CAM-ICU-defined delirium are limited. Rather than evaluating intradialytic hypotension solely as a binary variable based on its presence or absence, quantifying the hemodynamic burden in terms of cumulative hypotension duration and the frequency of hypotensive episodes may provide a more comprehensive and clinically informative approach to assessing neurological vulnerability.

Therefore, this study aimed to examine the association between intradialytic hemodynamic instability and CAM-ICU-defined delirium in critically ill patients receiving RRT in a tertiary ICU.

## 2. Materials and Methods

### 2.1. Study Design and Data Collection

This single-center, retrospective observational study was conducted to investigate the association between intradialytic hemodynamic instability and delirium in critically ill patients undergoing renal replacement therapy (RRT). Adult patients admitted to the tertiary intensive care unit (ICU) of Yalova Training and Research Hospital between September 2025 and May 2026 who underwent RRT during their ICU stay were evaluated for inclusion.

Demographic, clinical, laboratory, RRT-related, and hemodynamic data were retrospectively retrieved from the hospital electronic medical records and ICU monitoring records. Clinical variables included age, sex, presence of sepsis, requirement for mechanical ventilation, and duration of mechanical ventilation. The severity of critical illness was assessed using the Acute Physiology and Chronic Health Evaluation II (APACHE II) score, while the extent of organ dysfunction was evaluated using the Sequential Organ Failure Assessment (SOFA) score [18,19]. Hemodynamic variables included mean arterial pressure (MAP) recorded during RRT, the number of intradialytic hypotensive episodes, and the cumulative duration of MAP < 65 mmHg.

### 2.2. Study Population

All patients aged ≥18 years who were admitted to the ICU during the study period and received RRT during their ICU stay were assessed for eligibility.

Patients were eligible for inclusion if they met the following criteria: (i) age ≥ 18 years, (ii) admission to the tertiary ICU, (iii) receipt of RRT during the ICU stay, (iv) an ICU length of stay of at least 24 h, and (v) at least one CAM-ICU assessment recorded during the ICU stay, allowing ascertainment of delirium status.

Patients with a history of pre-diagnosed dementia, acute cerebrovascular event, severe traumatic brain injury, persistent coma, or primary psychotic disorder were excluded from the study. Patients without any documented CAM-ICU assessment, or with incomplete hemodynamic monitoring data precluding calculation of the study’s primary exposure variables, were also excluded.

### 2.3. Indications for Renal Replacement Therapy

Acute kidney injury was defined and staged according to the Kidney Disease: Improving Global Outcomes (KDIGO) Clinical Practice Guideline for Acute Kidney Injury [20]. RRT was initiated according to clinical indications routinely applied in our ICU, including refractory hyperkalemia, severe metabolic acidosis, fluid overload unresponsive to conservative management, and other clinically appropriate indications for kidney replacement therapy. All patients received intermittent hemodialysis (IHD) for AKI, and patients receiving maintenance hemodialysis before ICU admission were not included.

### 2.4. Assessment of Delirium

Delirium was assessed using the Confusion Assessment Method for the Intensive Care Unit (CAM-ICU), a validated instrument developed for the systematic assessment of delirium in critically ill patients, including those receiving mechanical ventilation [11]. CAM-ICU assessments were performed once daily as part of routine clinical practice by ICU physicians or nurses. The CAM-ICU evaluates four core features: (1) acute onset or fluctuating course of mental status changes, (2) inattention, (3) altered level of consciousness, and (4) disorganized thinking. A CAM-ICU assessment was considered positive according to the validated diagnostic algorithm, requiring the presence of Features 1 and 2 together with either Feature 3 or Feature 4. Thus, the presence of a single CAM-ICU feature alone was not considered sufficient for the diagnosis of delirium.

In this study, delirium was defined as at least one documented positive CAM-ICU assessment during the ICU stay; patients without any positive assessment were classified as CAM-ICU-negative (indicating the absence of a documented positive assessment rather than a confirmed, uninterrupted absence of delirium, as assessment availability during periods of deep sedation or temporary coma could not be retrospectively reconstructed). Patients with persistent coma were excluded from the study. Because CAM-ICU assessments were retrospectively obtained from routine clinical documentation, the dataset did not systematically capture sedation or consciousness level at each assessment or consistently identify observations that were unassessable because of deep sedation or coma. Therefore, assessment-level CAM-ICU eligibility with respect to sedation depth could not be retrospectively reconstructed.

### 2.5. Assessment of Intradialytic Hemodynamic Instability

Hemodynamic data recorded during RRT were retrospectively obtained from the available ICU and dialysis records. The dataset included the total number of RRT sessions performed during the ICU stay and the prescribed duration of individual dialysis sessions. However, the exact timing of individual RRT sessions relative to the first positive CAM-ICU assessment was not systematically recorded in the study dataset. Consequently, whether delirium preceded RRT initiation could not be reliably determined for every patient, precluding reconstruction of a patient-specific pre-delirium RRT exposure window. The hemodynamic variables were therefore analyzed as measures of intradialytic hemodynamic burden during the available RRT observation period rather than as exposures occurring exclusively before delirium onset.

Blood pressure was routinely measured at approximately 30 min intervals during RRT using noninvasive oscillometric monitoring, with additional measurements obtained when clinically indicated. Mean arterial pressure (MAP) values recorded during RRT were obtained from these routinely documented noninvasive blood pressure measurements and retrospectively retrieved from the ICU monitoring records. During RRT, intradialytic hypotension was defined as a MAP < 65 mmHg. A hypotensive episode was considered to begin when MAP decreased below 65 mmHg and to end when MAP subsequently returned to ≥65 mmHg. The interval between these two measurements was counted as hypotensive exposure; thus, if MAP was <65 mmHg at one measurement and ≥65 mmHg at the subsequent measurement, the entire intervening interval was included in the estimated hypotension duration. A subsequent decrease in MAP < 65 mmHg after documented recovery was considered a new episode. If MAP remained <65 mmHg at the last available measurement and no subsequent recovery measurement was documented before completion of the RRT session, the episode was considered to continue until the documented end of the session, and this interval was included in the cumulative hypotension duration. No predefined maximum interval was applied to irregularly spaced blood pressure measurements. No interpolation or imputation was performed for missing measurements, and calculations were based exclusively on the available documented blood pressure records.

The threshold of MAP < 65 mmHg was selected because it represents a widely used clinical threshold for hypotension and potentially inadequate organ perfusion in critically ill patients. Nevertheless, this threshold should be regarded as a pragmatic clinical definition rather than an individualized cerebral perfusion threshold, as autoregulatory requirements may differ among patients. To evaluate the association between hemodynamic instability and delirium, two primary exposure variables were defined: the cumulative duration of MAP < 65 mmHg during RRT and the number of intradialytic hypotensive episodes. The cumulative duration of MAP < 65 mmHg was estimated from consecutive recorded measurements and calculated as the total time during RRT in which MAP remained below the predefined threshold. For patients undergoing more than one RRT session, hemodynamic exposures were aggregated across the available RRT sessions. The number of intradialytic hypotensive episodes was defined as the total number of discrete hypotensive events meeting the predefined criteria.

### 2.6. Study Outcomes

The primary outcome was CAM-ICU-defined delirium during the ICU stay. Secondary outcomes included ICU mortality, duration of mechanical ventilation, and ICU length of stay. For the primary analysis, the cumulative duration of MAP < 65 mmHg and the number of intradialytic hypotensive episodes were considered the primary exposure variables, whereas CAM-ICU-defined delirium status was considered the dependent variable.

Given the retrospective observational design and the temporal resolution of routinely collected clinical data, the analyses were designed to evaluate the association between intradialytic hemodynamic instability and CAM-ICU-defined delirium rather than to establish a temporal or causal relationship between intradialytic hypotensive exposure and delirium onset.

### 2.7. Statistical Analyses

Statistical analyses were performed using IBM SPSS Statistics version 27.0 (IBM Corp., Armonk, NY, USA) and GraphPad Prism version 10.4.1 (GraphPad Software, LLC, Boston, MA, USA). The distribution of continuous variables was assessed using the Kolmogorov–Smirnov test together with visual inspection of the data. As continuous variables were not normally distributed, they were presented as median (Q1–Q3), whereas categorical variables were presented as number and percentage. Between-group comparisons were performed using the Mann–Whitney U test for continuous variables and Pearson’s chi-square test or Fisher’s exact test for categorical variables as appropriate according to expected cell frequencies. For continuous variables compared using the Mann–Whitney U test, effect size was calculated as the standardized effect size *r* = |Z|/√N, where Z represents the standardized Mann–Whitney U test statistic and N represents the total sample size. Cramer’s V was used as the effect-size measure for categorical variables.

Univariable binary logistic regression analyses were performed to evaluate the associations of intradialytic hypotension, cumulative duration of MAP < 65 mmHg, and number of hypotensive episodes with CAM-ICU positivity. Separate multivariable logistic regression models were subsequently constructed for each hemodynamic variable. The SOFA score obtained at ICU admission was used as the measure of baseline acute illness severity. Given the number of CAM-ICU-positive events and to reduce the risk of model overfitting, the number of predictors in each model was limited to four based on an approximately 10 events-per-variable approach. Accordingly, each hemodynamic variable was entered separately as the primary predictor and adjusted for age, sex, and admission SOFA score. All three multivariable models included the complete cohort of 87 patients. No data were missing for the variables included in these models; therefore, no imputation was performed. Logistic regression results were reported as odds ratios (ORs) with 95% confidence intervals (CIs).

The linearity of continuous predictors on the log-odds scale was assessed using Box–Tidwell-type transformation terms. No evidence of departure from linearity was observed for age, admission SOFA score, or number of hypotensive episodes; however, cumulative duration of MAP < 65 mmHg showed evidence of possible nonlinearity and was therefore interpreted cautiously. Potentially influential observations were assessed using Cook’s distance and standardized residuals. No observation had a Cook’s distance >1 or an absolute standardized residual >3.

Because a linear term assumes a constant change in the log-odds of CAM-ICU positivity per minute of hypotension, the odds ratio for cumulative MAP < 65 mmHg duration represents an averaged slope across the observed range and may not accurately reflect the association if the true relationship is nonlinear. A sensitivity analysis was therefore performed using three alternative functional forms of this variable, each adjusted for age, sex, and admission SOFA score: a log-transformed duration [log2(duration + 1)], a three-level categorical variable (0 min, 1–42 min, and ≥42.5 min, with 0 min as the reference), and a natural cubic spline with three degrees of freedom. Overall associations for the categorical and spline models were tested using likelihood-ratio tests.

To account for potential differences in the opportunity to accumulate hemodynamic exposure due to variation in the number of RRT sessions, an exploratory sensitivity analysis was additionally performed. For each patient, the cumulative duration of MAP < 65 mmHg and the total number of hypotensive episodes were normalized to the total number of RRT sessions, yielding MAP < 65 mmHg duration per RRT session and hypotensive episodes per RRT session, respectively. These normalized measures were compared between patients with and without delirium using the Mann–Whitney U test. This analysis was considered exploratory because the total number of RRT sessions reflected the entire ICU stay and could not be restricted to a patient-specific pre-delirium exposure period.

Receiver operating characteristic (ROC) curve analysis was performed to assess the discriminative ability of cumulative duration of MAP < 65 mmHg and the number of hypotensive episodes for CAM-ICU positivity. Areas under the curve (AUCs) with 95% CIs were calculated. Optimal cut-off values were determined by maximizing the Youden index, and the corresponding sensitivity and specificity values were reported.

Secondary clinical outcomes were compared according to CAM-ICU status. Duration of mechanical ventilation and ICU length of stay were compared using the Mann–Whitney U test, whereas ICU mortality was compared using Pearson’s chi-square test or Fisher’s exact test, as appropriate. All statistical tests were two-sided, and a *p*-value < 0.05 was considered statistically significant.

## 3. Results

A total of 99 patients were assessed for eligibility. Of these, 12 patients were excluded: six because of a pre-existing primary psychotic disorder, three because no CAM-ICU assessment was available during the ICU stay, and three because of incomplete hemodynamic data that precluded calculation of the primary exposure variables. Consequently, 87 patients were included in the final analysis, of whom 46 (52.9%) were classified as CAM-ICU-positive for delirium and 41 (47.1%) as CAM-ICU-negative (Figure 1).

Comparisons of demographic and clinical characteristics according to CAM-ICU status are presented in Table 1 and Figure 2. Age did not differ significantly between the groups, with a median age of 73.0 years (25th–75th percentile: 65.8–85.0) in CAM-ICU-positive patients and 77.0 years (70.5–86.0) in CAM-ICU-negative patients (*r* = 0.12, *p* = 0.247). In contrast, the proportion of female patients was significantly lower in the CAM-ICU-positive group than in the CAM-ICU-negative group (30.4% [14/46] vs. 53.7% [22/41], respectively; Cramer’s V = 0.24, *p* = 0.028).

Sepsis was more frequent among CAM-ICU-positive patients than among CAM-ICU-negative patients (73.9% vs. 61.0%); however, the difference did not reach statistical significance (Cramer’s V = 0.14, *p* = 0.197). Measures of critical illness severity were also comparable between the groups. Median APACHE II scores were 27.0 (16.8–36.5) and 28.0 (16.0–36.0) in the CAM-ICU-positive and CAM-ICU-negative groups, respectively (*r* = 0.05, *p* = 0.621). Similarly, median SOFA scores were 6.0 (4.0–8.0) and 5.0 (4.0–8.0), respectively, with no statistically significant between-group difference (*r* = 0.18, *p* = 0.087).

Regarding intradialytic hemodynamic parameters, intradialytic hypotension was more frequent among CAM-ICU-positive patients than among CAM-ICU-negative patients (76.1% [35/46] vs. 58.5% [24/41]); however, this difference did not reach statistical significance (Cramer’s V = 0.19, *p* = 0.080). In contrast, the cumulative duration of MAP < 65 mmHg was significantly longer in the CAM-ICU-positive group, with a median duration of 36.0 min (0.0–62.5) compared with 5.0 min (0.0–40.0) in the CAM-ICU-negative group (*r* = 0.21, *p* = 0.048). Similarly, the number of hypotensive episodes was significantly higher among CAM-ICU-positive patients (median, 3.0 [0.8–4.0]) than among CAM-ICU-negative patients (median, 1.0 [0.0–3.0]; *r* = 0.23, *p* = 0.032).

The requirement for mechanical ventilation was comparable between CAM-ICU-positive and CAM-ICU-negative patients (56.5% vs. 56.1%; Cramer’s V = 0.01, *p* = 0.968), with no significant difference in the duration of mechanical ventilation (median, 38.0 vs. 20.0 h; *r* = 0.09, *p* = 0.420). Likewise, ICU length of stay did not differ significantly between the groups (median, 7.0 vs. 5.0 days; *r* = 0.14, *p* = 0.186). ICU mortality was numerically higher among CAM-ICU-positive patients (78.3% vs. 65.9%), although the difference was not statistically significant (Cramer’s V = 0.14, *p* = 0.196).

Univariable binary logistic regression analyses showed that the presence of intradialytic hypotension (OR = 2.254, 95% CI: 0.899–5.651; *p* = 0.083), cumulative duration of MAP < 65 mmHg (OR = 1.004, 95% CI: 0.996–1.012; *p* = 0.296), and number of hypotensive episodes (OR = 1.152, 95% CI: 0.977–1.358; *p* = 0.092) did not show a statistically significant association with CAM-ICU positivity. These associations remained non-significant in separate multivariable models adjusted for age, sex, and disease severity as assessed by the SOFA score. The adjusted odds ratios were 2.109 (95% CI: 0.742–5.994; *p* = 0.161) for intradialytic hypotension, 1.004 (95% CI: 0.996–1.012; *p* = 0.341) for cumulative duration of MAP < 65 mmHg, and 1.122 (95% CI: 0.939– 1.340; *p* = 0.206) for the number of hypotensive episodes (Table 2). Adjustment for age, sex, and admission SOFA score did not materially alter the overall interpretation of the associations, and all 95% confidence intervals included unity. None of the three hemodynamic parameters showed a statistically significant association with CAM-ICU positivity in the adjusted models (Table 2).

The total number of RRT sessions performed during the ICU stay was higher in patients with CAM-ICU-defined delirium than in those without delirium (median, 3 [IQR, 2–4] vs. 2 [2,3] sessions; *p* = 0.017). The prescribed duration of individual dialysis sessions was also longer in patients with delirium (median, 4 [IQR, 3–4] vs. 3 [3,4] h; *p* = 0.027). Given these differences in RRT exposure, an exploratory per-session sensitivity analysis was performed. After normalization to the total number of RRT sessions, the median duration of MAP < 65 mmHg was 12.9 [IQR, 0.05–22.5] min/session in patients with delirium and 3.0 [0–20.0] min/session in those without delirium (*p* = 0.153). The corresponding number of hypotensive episodes was 1.0 [0.35–1.48] versus 0.9 [0–1.0] episodes/session, respectively (*p* = 0.249). Thus, the between-group differences in the cumulative hemodynamic measures were no longer statistically significant when normalized to the number of RRT sessions.

Receiver operating characteristic (ROC) curve analysis demonstrated that the cumulative duration of MAP < 65 mmHg had limited discriminative ability for CAM-ICU positivity, with an area under the curve (AUC) of 0.62 (95% CI: 0.50–0.74; *p* = 0.052). Based on the Youden index, the exploratory optimal cut-off value was ≥42.5 min, yielding a sensitivity of 45.7% and a specificity of 78.0%. The number of hypotensive episodes showed a statistically significant, although similarly modest, discriminative performance, with an AUC of 0.63 (95% CI: 0.51–0.75; *p* = 0.036). The optimal cut-off was ≥3 hypotensive episodes, corresponding to a sensitivity of 52.2% and a specificity of 70.7% (Table 3, Figure 3). The AUC values for both hemodynamic parameters were within the 0.60–0.65 range (Table 3, Figure 3).

Secondary clinical outcomes did not differ significantly according to CAM-ICU status. ICU mortality was numerically higher among CAM-ICU-positive patients, and ICU length of stay and duration of mechanical ventilation were numerically longer; however, none of these between-group differences reached statistical significance (Table 1).

In the sensitivity analysis using alternative functional forms of cumulative MAP < 65 mmHg duration, none of the models showed a statistically significant association with CAM-ICU positivity. The adjusted OR per doubling of log-transformed duration was 1.139 (95% CI: 0.963–1.345; *p* = 0.128). In the categorical model, compared with patients without hypotension, the adjusted OR was 1.028 (95% CI: 0.312–3.387; *p* = 0.964) for 1–42 min and 3.094 (95% CI: 0.938–10.211; *p* = 0.064) for ≥42.5 min (overall *p* = 0.090). The natural cubic spline model was also not significant (overall *p* = 0.115; test of nonlinearity *p* = 0.066). Although the categorical estimates suggested a possible threshold pattern, the confidence intervals were wide, and the cut-off was derived from the study data; this finding should therefore be considered hypothesis-generating (Table 4).

Of 99 patients assessed for eligibility, 12 were excluded (six for a pre-existing primary psychotic disorder, three for lack of a CAM-ICU assessment during the ICU stay, and three for incomplete hemodynamic data precluding calculation of the primary exposure variables). The remaining 87 patients were included in the final analysis, of whom 46 (52.9%) were classified as CAM-ICU-positive for delirium and 41 (47.1%) as CAM-ICU-negative.

## 4. Discussion

The principal finding of the present study was that critically ill patients with delirium experienced a greater cumulative intradialytic hemodynamic burden than those without delirium—reflected both in the total time spent with a mean arterial pressure (MAP) < 65 mmHg and in the number of discrete hypotensive episodes during renal replacement therapy (RRT). These findings highlight the potential value of quantitative hemodynamic measures, rather than relying solely on a binary definition of intradialytic hypotension, to more comprehensively characterize the physiological burden that may be associated with acute neurocognitive dysfunction. The median cumulative duration of MAP < 65 mmHg was 36 min in patients with delirium compared with 5 min in those without delirium (*p* = 0.048). Although this between-group difference reached the conventional threshold for statistical significance, the *p*-value was borderline, and the corresponding association was not statistically significant in either the univariable or adjusted logistic regression analysis; therefore, this finding should be interpreted cautiously. Similarly, the number of hypotensive episodes was significantly higher in the delirium group (median, 3 vs. 1; *p* = 0.032). Taken together, these observations suggest that quantitative measures of hypotensive burden, including its duration and recurrence, may provide additional information beyond the binary presence or absence of intradialytic hypotension, although their temporal and independent relationship with delirium remains uncertain.

The prevalence of delirium, assessed using the Confusion Assessment Method for the Intensive Care Unit (CAM-ICU), was 52.9% in our cohort. Delirium has been reported in approximately 20–40% of general ICU populations, with substantially higher rates of up to 60–80% among mechanically ventilated patients [1,21,22,23]. The relatively high prevalence observed in our study may reflect the substantial burden of organ dysfunction in a cohort composed entirely of critically ill patients requiring RRT. This interpretation is supported by previous evidence linking renal dysfunction to delirium: Jäckel et al. reported delirium in 60.1% of ICU patients with AKI and identified advanced AKI stage, older age, mechanical ventilation, and severe shock as independent risk factors [4]. These findings support the concept that critically ill patients with renal dysfunction, particularly those requiring RRT, represent a population at high risk for delirium.

Delirium is a multifactorial syndrome resulting from the interaction between predisposing vulnerability and acute precipitating factors. Advanced age, pre-existing cognitive impairment, frailty, and comorbid disease may increase baseline susceptibility, whereas acute illness, infection and systemic inflammation, metabolic and electrolyte disturbances, sedative or psychoactive medication exposure, sleep disruption, pain, and immobility may act as precipitating factors. In critically ill patients, these factors frequently coexist and may interact with hemodynamic instability to further increase vulnerability to acute brain dysfunction. Although our cohort consisted predominantly of older patients, age did not differ significantly between the CAM-ICU-positive and CAM-ICU-negative groups, suggesting that the observed difference in delirium status cannot be attributed to age alone. Accordingly, intradialytic hypotension should be interpreted within this broader multifactorial framework rather than as an isolated determinant of delirium.

Known risk factors for delirium in the ICU include advanced age, greater illness severity, sepsis, coma, benzodiazepine exposure, and mechanical ventilation, many of which reflect the severity of the underlying critical illness. Consistent with this concept, patients with and without delirium in our cohort were comparable with respect to age, SOFA scores, sepsis, and the frequency of mechanical ventilation. However, the absence of statistically significant between-group differences in these variables does not exclude their potential confounding effects. Nevertheless, given the observational nature of the study, the potential influence of unmeasured residual confounding cannot be completely excluded.

Mechanical ventilation is widely recognized as a potential contributor to delirium in critically ill patients. This association may partly reflect greater underlying illness severity, but it may also arise from interventions accompanying ventilatory support, particularly the use of sedative and analgesic medications. Studies focusing specifically on mechanically ventilated populations have also linked delirium to longer periods of ventilatory support [6,23]. In the present cohort, however, the proportion of patients requiring mechanical ventilation was nearly the same in the CAM-ICU-positive and CAM-ICU-negative groups (56.5% vs. 56.1%, *p* = 0.968). Although patients with delirium had a numerically longer median duration of mechanical ventilation (38 vs. 20 h), the difference did not reach statistical significance (*p* = 0.420). Several characteristics of our RRT cohort may account for the divergence from previous findings, including relatively uniform illness severity, insufficient statistical power to detect modest differences, and residual confounding related to sedation exposure. The association should also be considered potentially bidirectional. Mechanical ventilation and the treatments accompanying it may predispose patients to delirium, while the presence of delirium may, in turn, interfere with successful ventilator liberation and thereby extend the duration of mechanical ventilatory support.

Among demographic and baseline clinical characteristics, the only statistically significant between-group difference was sex distribution, with a lower proportion of women among patients with delirium (*p* = 0.028). However, the role of sex in delirium risk remains uncertain, with previous studies reporting no consistent independent association between sex and delirium [24,25]. Given the limited sample size and the secondary nature of this finding, this association should be interpreted cautiously.

Although cumulative MAP < 65 mmHg duration and the number of hypotensive episodes differed significantly between CAM-ICU groups in the unadjusted between-group comparisons, none of the hemodynamic exposures reached statistical significance in either the univariable or multivariable logistic regression models. Adjustment for age, sex, and admission SOFA score did not materially alter the overall interpretation of these associations. These findings are not necessarily contradictory, as the Mann–Whitney U test and logistic regression address different statistical questions: the former evaluates differences in the distribution of continuous variables between two groups, whereas the latter estimates the association of each exposure with the odds of CAM-ICU positivity. Accordingly, the observed between-group differences should not be interpreted as evidence of an independent association with delirium.

An additional consideration is the unequal opportunity for accumulation of intradialytic hemodynamic exposure between the groups. Patients with delirium underwent a greater number of RRT sessions and had longer prescribed dialysis-session durations than those without delirium. In the exploratory sensitivity analysis, the between-group differences in MAP < 65 mmHg duration and hypotensive episode count were no longer statistically significant after normalization to the total number of RRT sessions. These findings suggest that the differences observed in the cumulative hemodynamic measures may have been influenced, at least in part, by unequal RRT exposure opportunity. However, because the total number of RRT sessions encompassed the entire ICU stay rather than a specifically defined pre-delirium period, this sensitivity analysis does not resolve the temporal limitation of the retrospective dataset and should therefore be interpreted as exploratory.

In our cohort, ICU mortality was numerically higher among patients with delirium than among those without delirium (78.3% vs. 65.9%), accompanied by longer durations of mechanical ventilation (median, 38.0 vs. 20.0 h) and ICU stay (median, 7.0 vs. 5.0 days); however, none of these differences reached statistical significance. These trends are directionally consistent with previous studies linking delirium to increased mortality and prolonged hospitalization [10,22]. The absence of statistical significance should be interpreted cautiously, as the limited sample size, high overall illness severity, and substantial mortality in both groups may have reduced the statistical power to detect an additional prognostic effect of delirium. Thus, our findings do not establish a statistically significant association between delirium and these adverse clinical outcomes in this high-risk cohort.

A major strength of our study is its focus on the relationship between delirium and intradialytic hemodynamic instability, a clinically relevant aspect of RRT. Additional strengths include the quantitative assessment of hemodynamic instability through the duration of time spent with MAP < 65 mmHg and the number of hypotensive episodes, rather than relying solely on the presence or absence of IDH, as well as the use of multivariable models adjusted for age, sex, and admission SOFA score. Although the association between AKI and delirium is well established, most prior studies in this area have examined AKI as a categorical exposure or have focused on stable chronic hemodialysis populations without directly quantifying intradialytic hemodynamic parameters in critically ill patients receiving RRT; few studies have directly and quantitatively evaluated this specific relationship in this population.

Our study has several limitations. First, its retrospective, single-center design and relatively small sample size may limit the generalizability of the findings and reduce statistical power, particularly for multivariable analyses. Second, the observational nature of the study precludes causal inference, and residual confounding from unmeasured or incompletely characterized factors cannot be excluded. Sedation-related confounding is particularly relevant because sedative and analgesic exposure may influence both hemodynamic stability during RRT and the risk of delirium.

Third, delirium assessment was based on once-daily CAM-ICU evaluations performed as part of routine clinical practice. Given the fluctuating nature of delirium, this assessment frequency may have resulted in underdetection or imprecise identification of delirium onset.

Fourth, the temporal relationship between RRT exposure and delirium could not be precisely established. The exact timing of individual RRT sessions relative to the first positive CAM-ICU assessment was not systematically captured in the study dataset; consequently, patient-specific pre-delirium exposure windows and cumulative pre-delirium RRT hours could not be reliably reconstructed. In addition, patients with delirium underwent a greater number of RRT sessions during their ICU stay, potentially providing greater opportunity to accumulate hypotensive episodes and time with MAP < 65 mmHg. Thus, cumulative measures of intradialytic hypotension may partly reflect differences in RRT exposure opportunity rather than solely differences in hemodynamic vulnerability.

Fifth, intradialytic blood pressure measurements were obtained retrospectively from routine clinical records. Measurement intervals may therefore have varied between patients and across RRT sessions and may have become more frequent following recognition of hemodynamic instability, potentially affecting estimates of both hypotensive episode frequency and cumulative hypotension duration. Moreover, because hypotensive duration was estimated from consecutive intermittent measurements, assigning the interval to the first documented recovery measurement to hypotensive exposure may have overestimated the true duration of individual episodes, particularly when recording intervals were prolonged or irregular. In addition, ultrafiltration volume and rate could not be consistently retrieved from the retrospective records and therefore could not be incorporated into the analyses, precluding evaluation of their potential contribution to intradialytic hemodynamic instability.

Accordingly, cumulative time with MAP < 65 mmHg and the number of hypotensive episodes should be interpreted as estimates of hemodynamic burden derived from routinely recorded intermittent measurements rather than as continuous measures of blood pressure exposure. Furthermore, no formal adjustment for multiple comparisons, such as false discovery rate correction, was applied; therefore, the nominally significant between-group findings should be interpreted cautiously and regarded as exploratory.

These findings therefore require validation in larger, multicenter, prospective studies with more detailed assessment of hemodynamic, sedation-related, and neurological parameters.

## 5. Conclusions

In this study, critically ill patients with CAM-ICU-defined delirium had a longer cumulative duration of MAP < 65 mmHg and a greater number of intradialytic hypotensive episodes than patients without delirium. However, the corresponding regression estimates were imprecise and did not reach statistical significance in either univariable or adjusted models. These findings identify a potential relationship between intradialytic hemodynamic burden and delirium but do not establish an independent or causal association. Larger prospective studies with standardized hemodynamic monitoring and temporally detailed delirium assessment are warranted.

## Figures and Tables

**Figure 1:**
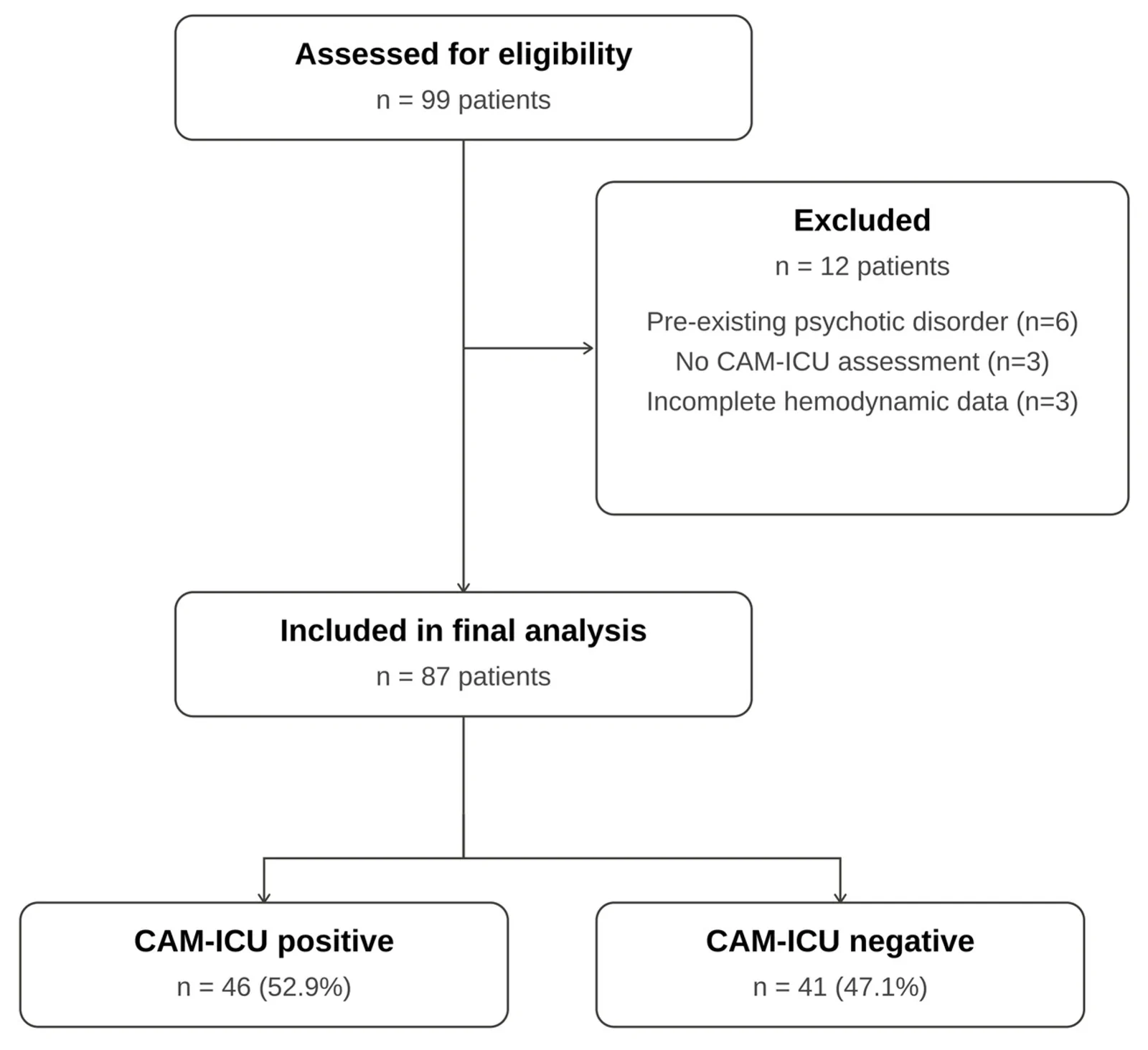
Patient flow diagram.

**Figure 2:**
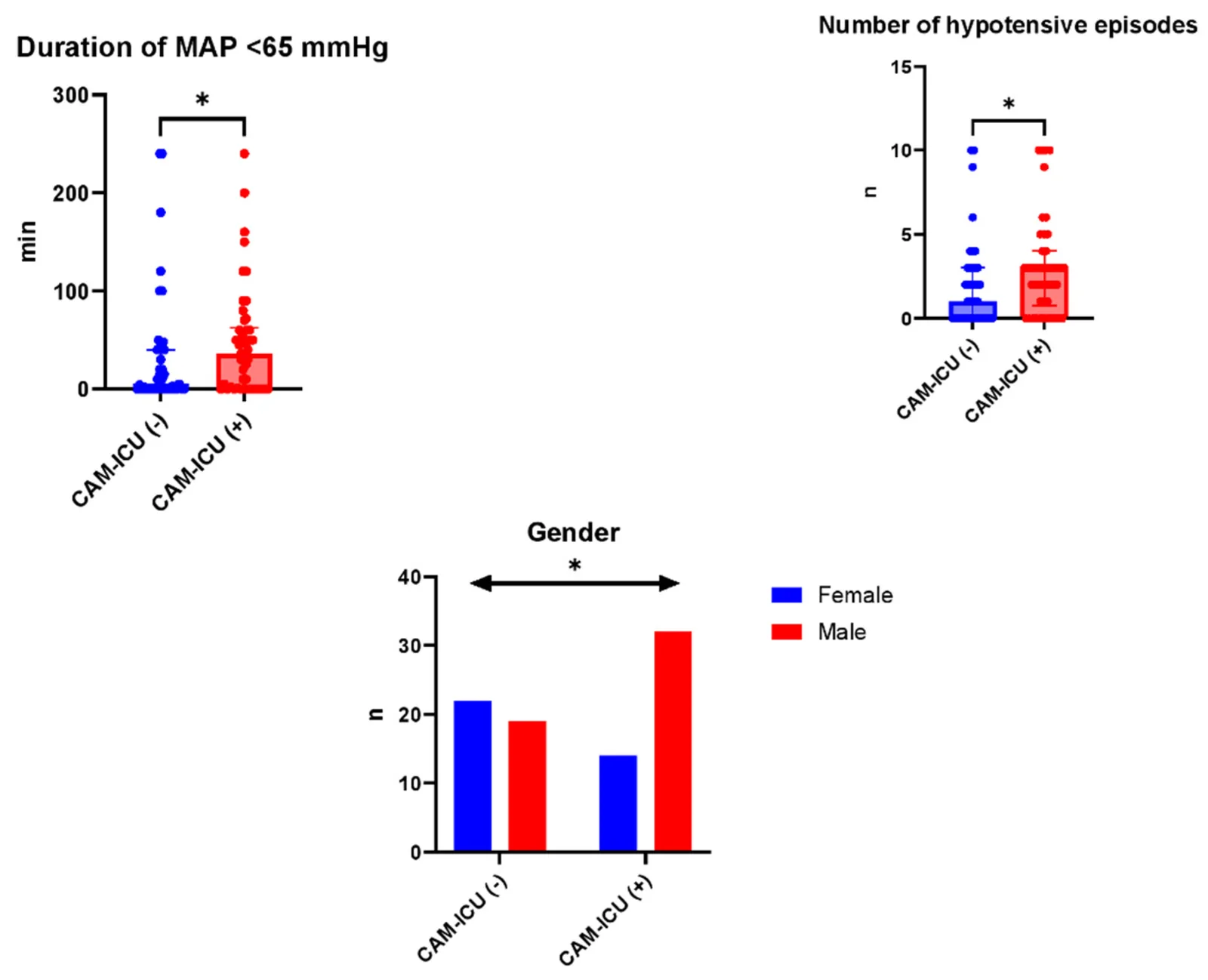
Comparison of demographic and hemodynamic parameters according to CAM-ICU status. Continuous hemodynamic variables are presented as medians and interquartile ranges (IQRs), with boxes representing the IQR and central lines indicating the median. Individual dots represent individual patient values. Sex distribution is presented as patient counts. The figure illustrates the between-group differences in cumulative MAP < 65 mmHg duration, number of hypotensive episodes, and sex distribution. * *p* < 0.05 indicates statistical significance. Abbreviations: CAM-ICU: Confusion Assessment Method for the Intensive Care Unit, MAP: Mean Arterial Pressure.

**Figure 3:**
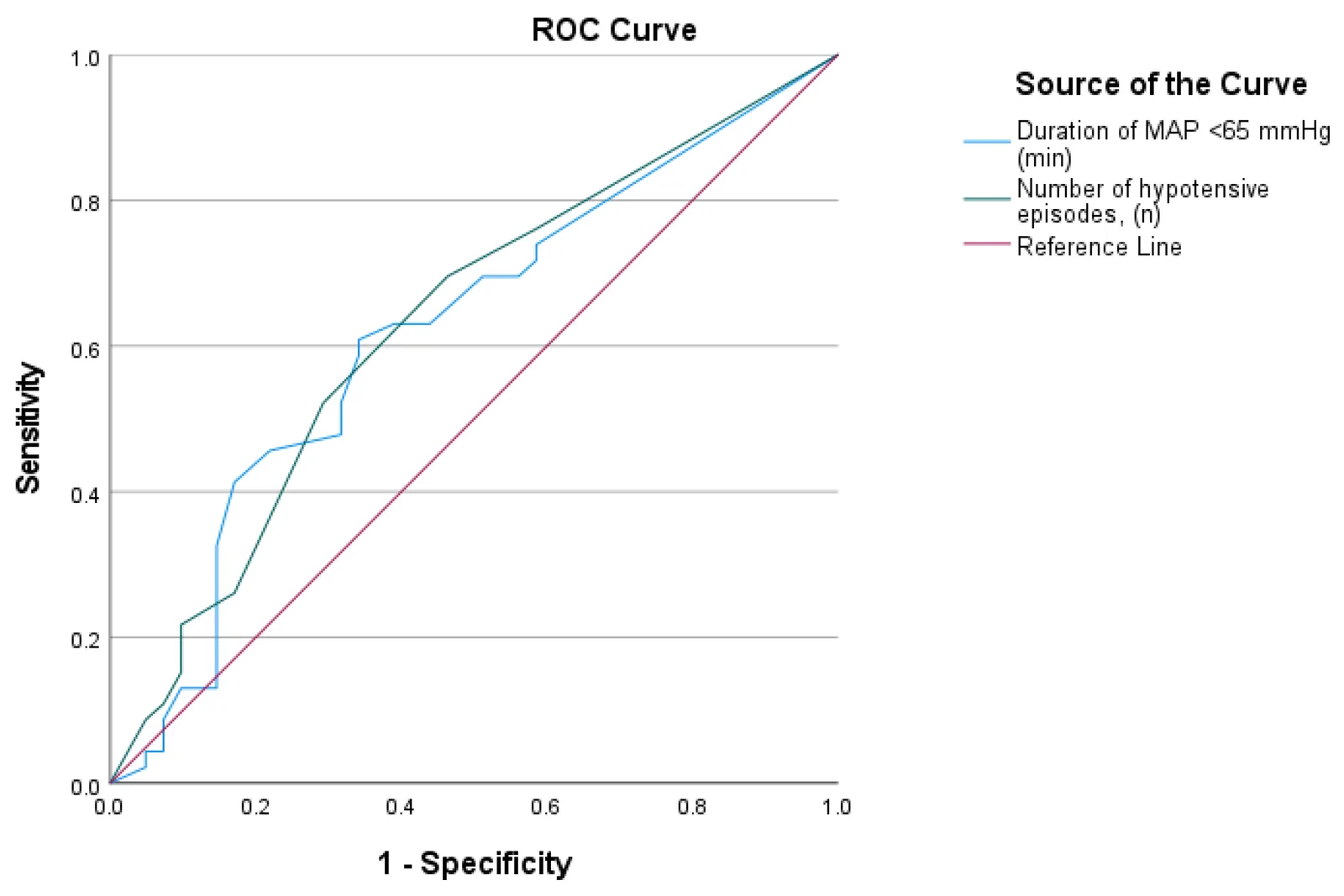
Receiver operating characteristic curves of hemodynamic parameters for discriminating CAM-ICU positivity. ROC curves showing the discriminative performance of cumulative duration of MAP < 65 mmHg and the number of hypotensive episodes for CAM-ICU positivity. The AUC was 0.62 (95% CI, 0.50–0.74; *p* = 0.052) for cumulative MAP < 65 mmHg duration and 0.63 (95% CI, 0.51–0.75; *p* = 0.036) for the number of hypotensive episodes. **Abbreviations**: ROC: Receiver Operating Characteristic, CAM-ICU: Confusion Assessment Method for the Intensive Care Unit, MAP: Mean Arterial Pressure.

**Table 1:** Demographic and clinical characteristics of patients according to CAM-ICU status.

	All Patients N = 87	CAM-ICU (+) N = 46	CAM-ICU (−) N = 41	Effect Size (*r* or Cramer’s V)	*p*-Value
Age (years) ^a^	76.0 (69.0–85.0)	73.0 (65.8–85.0)	77.0 (70.5–86.0)	0.12	0.247
Sex, (F) ^b^	36 (41.4)	14 (30.4)	22 (53.7)	0.24	0.028
Sepsis ^b^	59 (67.8)	34 (73.9)	25 (61.0)	0.14	0.197
APACHE II score ^a^	28.0 (16.0–36.0)	27.0 (16.8–36.5)	28.0 (16.0–36.0)	0.05	0.621
SOFA score ^a^	6.0 (4.0–8.0)	6.0 (4.0–8.0)	5.0 (4.0–8.0)	0.18	0.087
Intradialytic hypotension ^b^	59 (67.8)	35 (76.1)	24 (58.5)	0.19	0.080
Duration of MAP < 65 mmHg (min) ^a^	20.0 (0.0–50.0)	36.0 (0.0–62.5)	5.0 (0.0–40.0)	0.21	0.048
Number of hypotensive episodes ^a^	2.0 (0.0–3.0)	3.0 (0.8–4.0)	1.0 (0.0–3.0)	0.23	0.032
Mechanical ventilation ^b^	49 (56.3)	26 (56.5)	23 (56.1)	0.01	0.968
Duration of mechanical ventilation (hours) ^a^	20.0 (0.0–72.0)	38.0 (0.0–76.5)	20.0 (0.0–70.0)	0.09	0.420
ICU length of stay (days) ^a^	6.0 (3.0–9.0)	7.0 (4.0–10.0)	5.0 (3.0–8.5)	0.14	0.186
ICU mortality ^b^	63 (72.4)	36 (78.3)	27 (65.9)	0.14	0.196

a: median (25–75%), b: *n* (%). Data are presented as median (25th–75th percentile) for continuous variables and *n* (%) for categorical variables. Effect sizes for continuous variables were calculated as standardized r (|Z|/√N) from the Mann–Whitney U test, whereas Cramer’s V was used for categorical variables. Between-group comparisons were performed using the Mann–Whitney U test for continuous variables and Pearson’s chi-square or Fisher’s exact test for categorical variables, as appropriate. A *p*-value < 0.05 was considered statistically significant. **Abbreviations:** CAM-ICU: Confusion Assessment Method for the Intensive Care Unit, APACHE II: Acute Physiology and Chronic Health Evaluation II, SOFA: Sequential Organ Failure Assessment, MAP: Mean Arterial Pressure, ICU: Intensive Care Unit, F: Female.

**Table 2:** Univariable and adjusted logistic regression analyses of hemodynamic factors associated with CAM-ICU positivity.

	Univariable		Multivariable (Adjusted *)	
	OR (95% CI)	*p*-Value	OR (95% CI)	*p*-Value
Intradialytic hypotension	2.254 (0.899–5.651)	0.083	2.109 (0.742–5.994)	0.161
Cumulative MAP < 65 mmHg duration (min)	1.004 (0.996–1.012)	0.296	1.004 (0.996–1.012)	0.341
Number of hypotensive episodes, (*n*)	1.152 (0.977–1.358)	0.092	1.122 (0.939–1.340)	0.206

*: Each hemodynamic variable was evaluated in a separate multivariable logistic regression model adjusted for age, sex, and SOFA score at ICU admission. All models included 87 patients. ORs are presented with 95% CIs. Odds ratios (ORs) and 95% confidence intervals (CIs) were estimated using binary logistic regression. A *p*-value < 0.05 was considered statistically significant. **Abbreviations**: OR: Odds Ratio, CI: Confidence Interval, MAP: Mean Arterial Pressure.

**Table 3:** Receiver operating characteristic analysis of hemodynamic parameters for discriminating CAM-ICU positivity.

	AUC (95% CI)	*p*-Value	Optimal Cut-Off	Sensitivity (%)	Specificity (%)
Duration of MAP < 65 mmHg (min)	0.62 (0.50–0.74)	0.052	≥42.5	45.7	78.0
Number of hypotensive episodes, (*n*)	0.63 (0.51–0.75)	0.036	≥3	52.2	70.7

Receiver operating characteristic (ROC) analysis was used to evaluate the discriminative performance of cumulative duration of MAP < 65 mmHg and the number of hypotensive episodes for CAM-ICU positivity. Optimal cut-off values were determined using the Youden index. AUC values are presented with 95% confidence intervals together with the corresponding sensitivity and specificity. **Abbreviations**: AUC: Area Under the Curve, CI: Confidence Interval, MAP: Mean Arterial Pressure, CAM-ICU: Confusion Assessment Method for the Intensive Care Unit.

**Table 4:** Sensitivity analysis of alternative functional forms of cumulative MAP < 65 mmHg duration and CAM-ICU positivity.

Functional Form of MAP < 65 mmHg Duration	CAM-ICU (+)/*n*	Adjusted OR (95% CI) *	*p*-Value
Linear, per 1 min (primary model)	46/87	1.004 (0.996–1.012)	0.341
Log-transformed, per doubling of log_2_(duration + 1)	46/87	1.139 (0.963–1.345)	0.128
Categorical			0.090 †
0 min	12/29	1.00 (reference)	–
1–42 min	13/28	1.028 (0.312–3.387)	0.964
≥42.5 min	21/30	3.094 (0.938–10.211)	0.064
Natural cubic spline (3 df), overall	46/87	–	0.115 †
Test of nonlinearity (spline vs. linear)		–	0.066 †

(*): Each functional form was evaluated in a separate binary logistic regression model adjusted for age, sex, and SOFA score at ICU admission. All models included 87 patients. (†): Likelihood-ratio test. The ≥42.5 min category boundary corresponds to the Youden-derived cut-off in Table 3 and was therefore derived from the study data. A *p*-value < 0.05 was considered statistically significant. **Abbreviations**: CAM-ICU: Confusion Assessment Method for the Intensive Care Unit, CI: Confidence Interval, df: degrees of freedom, MAP: Mean Arterial Pressure, OR: Odds Ratio, SOFA: Sequential Organ Failure Assessment.

## Data Availability

The data presented in this study are available from the corresponding author upon reasonable request. The data are not publicly available due to privacy and ethical restrictions related to the protection of patient confidentiality.
